# Effects and mode of action of chitosan and ivy fruit saponins on the microbiome, fermentation and methanogenesis in the rumen simulation technique

**DOI:** 10.1093/femsec/fiv160

**Published:** 2015-12-16

**Authors:** Alejandro Belanche, Eric Pinloche, David Preskett, C. Jamie Newbold

**Affiliations:** 1 Institute of Biological, Environmental and Rural Sciences, Aberystwyth University, SY23 3DA, Aberystwyth, UK; 2 BioComposites Centre, Bangor University, LL57 2UW, Bangor, UK

**Keywords:** chitosan, feed additive, ivy fruit saponins, methane, rumen fermentation

## Abstract

This study investigates the effects of supplementing a control diet (CON) with chitosan (CHI) or ivy fruit saponins (IVY) as natural feed additives. Both additives had similar abilities to decrease rumen methanogenesis (–42% and –40%, respectively) using different mechanisms: due to its antimicrobial and nutritional properties CHI promoted a shift in the fermentation pattern towards propionate production which explained about two thirds of the decrease in methanogenesis. This shift was achieved by a simplification of the structure in the bacterial community and a substitution of fibrolytic (Firmicutes and Fibrobacteres) by amylolytic bacteria (Bacteroidetes and Proteobacteria) which led to greater amylase activity, lactate and microbial protein yield with no detrimental effect on feed digestibility. Contrarily, IVY had negligible nutritional properties promoting minor changes in the fermentation pattern and on the bacterial community. Instead, IVY modified the structure of the methanogen community and decreased its diversity. This specific antimicrobial effect of IVY against methanogens was considered its main antimethanogenic mechanism. IVY had however a negative impact on microbial protein synthesis. Therefore, CHI and IVY should be further investigated *in vivo* to determine the optimum doses which maintain low methanogenesis but prevent negative effects on the rumen fermentation and animal metabolism.

## Abstract (precis)

Rumen function is generally suboptimal leading to loses in the form of methane and nitrogen, analysis of the rumen microbiome is vital to understand the mode of action of new feed additives to improve rumen function.

## INTRODUCTION

Methane (CH_4_) is one of the three main greenhouse gases and its global warming potential is 25-fold higher than CO_2_ (Gill, Smith and Wilkinson 2010). Agriculture is responsible for about half of total anthropogenic methane emissions, of which 32% comes from enteric fermentation in ruminants (Broucek 2014). A number of chemical feed additives, antibiotics, methane inhibitors, defaunating agents and plant extracts have been shown to decrease methane emissions and to improve animal performance (Patra 2010). However, concerns over the presence of chemical residues in animal products, the development of bacterial resistance to antibiotics and the excessive toxicity and cost of some plant extracts have limited their utilization in animal nutrition (Wina, Muetzel and Becker 2005). Thus, the scientific community is still actively seeking alternative feed additives that could improve rumen function (Soliva *et al.*^2011^).

Here, we investigate two novel natural compounds which have antimicrobial properties and could theoretically be used to manipulate the rumen microbial ecosystem: Chitosan (*CHI)* is the second most abundant natural biopolymer on earth after the cellulose and is commonly found in the shells of marine crustaceans and the cell walls of fungi. As a non-toxic, biodegradable carbohydrate polymer, CHI has received attention for a diverse range of potential applications in medicine and food preservations due to its antimicrobial properties against bacteria, moulds and yeast (Kong *et al.*^2010^). Several studies have reported potential benefits of CHI on the rumen fermentation and methanogenesis (Goiri, Garcia-Rodriguez and Oregui 2009a,b; Goiri, Oregui and Garcia-Rodriguez 2010). However, the mechanisms of action of CHI on the rumen microbial ecosystem still need to be assessed prior to its commercial utilization as a feed additive.

Saponins are a group of plant secondary metabolites that form stable foams in aqueous solutions similar to soap, hence the name ‘saponin’. Chemically, saponins include compounds that are glycosylated steroids, triterpenoids and steroid alkaloids. Some of these saponins have been shown to modify rumen fermentation and enhance animal production (Wina, Muetzel and Becker 2005). So far, most studies have focused on saponins extracts obtained from *Yucca schidigera*, *Quillaja saponaria*, *Acacia auriculiformins*, *Sapindus saponaria*, *Sesbania sesban* and *Medicago sativa* (Patra and Saxena 2009a,b), and to our knowledge there are no studies describing the effect of saponins from ivy fruit (*IVY*, *Hedera helix*) as a feed additive.

In a previous experiment, we observed that CHI and IVY were able to decrease protozoal activity and to modify rumen fermentation when incubated in batch cultures at concentrations above 1 g L^−1^ (Belanche, Ramos-Morales and Newbold 2015). However, batch cultures are unable to sustain a stable fermentation pattern over multiple days due to the accumulation of end-products and eventually the whole microbial community collapses. The rumen simulation technique (Rusitec) enables a long-term and stable *in vitro* fermentation to be maintained for several weeks, a time-scale sufficient to allow the study of the possible long-term adaptation that may occur (Patra and Saxena 2009a). Therefore, this paper aims to expand knowledge on the activity and mode of action of CHI and IVY on rumen function and methanogenesis. A multiomics approach was adopted based on a detailed description of the rumen fermentation, enzymatic activity, microbial protein synthesis, as well as a thorough characterization of the bacterial and methanogens communities using Next Generation Sequencing.

## MATERIALS AND METHODS

### Apparatus and diets

Rusitec was used (Czerkawski and Breckenridge 1977) to investigate the effect of a control diet alone (*CON*) supplemented (5% inclusion rate in DM) with either soluble chitosan (*CHI*) or an ivy fruit extract (*IVY*) rich in saponins. CHI was >85% deacetylated with a viscosity equal to 140 mPas in 1% acetic acid solution at 25ºC (Nitta Gelatin India Ltd. Cocin, Kerala, India). IVY was obtained by extraction of raw organic compounds from ivy fruit meal using ethanol followed by a diethyl ether defatting step resulting in an extract with 15% saponins content in DM. The inclusion rate used in this experiment were chosen based on previous dose–response studies which noted that CHI and IVY can modify the rumen fermentation when incubated at concentrations above 1g L^−1^ in batch cultures (Belanche, Ramos-Morales and Newbold 2015). Experimental diets had a 50:50 forage-to-concentrate ratio, and all their ingredients were ground to pass through 1mm^2^ sieve size (Table S1, Supporting Information).

Animal procedures were carried out according to the Home Office Scientific Procedures, Act 1986 (PLL 40/3653; PIL 40/9798). Rumen fluid was obtained from four barren rumen-cannulated Holsten–Frisian cows fed at maintenance (diet composed of 67% perennial ryegrass hay and 33% concentrate, on a DM basis). Rumen contents were sampled before the morning feeding, filtered and stored anaerobically at 39ºC.

The trial consisted of a single incubation period using 12 vessels which were considered as experimental units. Thus, each dietary treatment had four replicates which were randomly allocated to the vessels and inoculated with rumen fluid from different cows. Vessels had an effective volume of 800 ml and were kept at 39ºC under permanent vertical agitation. On day 1, vessels were inoculated with strained rumen fluid diluted 1:1 with artificial saliva (McDougall 1948), then artificial saliva was continuously infused at a rate of 640 ml d^−1^ (dilution rate of 3.33% h^−1^) using a multichannel peristaltic pump (Watson–Marlow 200 series, Cornwall, UK). Squeezed rumen solids (60g FM) were placed in nylon bags (110 × 60 mm, pore size 100 μm^2^) and incubated in each vessel for 1day to provide solid-associated bacteria, while experimental feed was supplied in a second bag. On subsequent days, the bag that had remained 2 days in each vessel was squeezed, washed with 30ml of artificial saliva. The liquid fraction of the washing was returned to the vessels, and a new feed bag was inserted containing 20g DM.

### Experimental procedure and sampling

The incubation trial consisted of 18 days, using the first 10 days for adaptation and the last 8 for sampling. Dry matter degradation, methane emissions and outflow of fermentation products were measured on days 11, 12, 13 and 14. Nylon bags were collected, rinsed with cold water for 20 min, and DM disappearance after 48 h incubation was calculated from the loss in weight. Fermentation gases were collected in gas-tight bags (TECOBAG 5L, PETP/AL/PE-12/12/75, Tesseraux container GmbH, Germany) to measure total gas and methane production (ATI Unicam 610 Series, Gas Chromatograph, UK). Daily production of ammonia and VFA were measured in the overflow flasks with 10ml of saturated HgCl_2_ (diluted 1:5) added to stop the fermentation.

To describe diurnal changes in the fermentation pattern, on days 15, 16 and 17 fluid from within vessels was sampled (15 mL) by aspiration at 2, 4, 8 and 24 h after feeding. The pH was immediately recorded, and each sample was divided into four subsamples. The first subsample (10 mL) was snap frozen in liquid N for microbial characterization and enzymatic activity. The second subsample (1.6 mL) was diluted with 0.4 mL of deproteinising solution (20% ortophosporic acid containing 10 mM of 2-ethilbutyric acid) for VFA determination. The third subsample (0.8 mL) was diluted with 0.4 mL of trichloro-acetate (25% wt:vol) for ammonia analysis, and the fourth subsample (0.8 mL) was snap frozen for lactate determination.

Microbial protein synthesis was measured using ^15^N was as microbial marker (Carro and Miller 1999). On day 12, each vessel was infused with 3 mg of ^15^N, as (^15^NH_4_)_2_SO_4_ to label the ammonia-N pool. From day 12 onwards, ^15^N was added to the artificial saliva (3.7 mg L^−1^) to label the microbial protein. On days 15, 16 and 17 residues of the feed bags were daily mixed with their associated effluents, homogenized in a blender at low speed for 1 min and pooled to reconstitute the total digesta. One portion of total digesta (100 g) was frozen to generate the non-ammonia N (NAN) fraction, while other portion (200g) was used to isolate the total bacteria and the ammonia-N fractions (Belanche *et al.*^2013^).

To evaluate the antimicrobial effect of CHI and IVY against pathogens, on the last day of the experiment (18 days), each vessel was inoculated with 5 mL of non-toxigenic *Escherichia coli* O157:H7 strain (NCTC 12900) grown for 2 days in liquid media (LB Broth, Life Technologies, UK). Then, serial samples (1 mL) were taken at 0, 3, 6, 9 and 24 h post-inoculation. *Escherichia coli* survival was measured by plating 20 μL of sample on to MacConkey agar base Nº 3 (Oxoid Ltd, Basingstoke, Hampshire, UK) containing rifampicin (50 μg mL^−1^) and incubating at 37°C for 24 h prior to counting.

### Sample analyses

Feed chemical composition, methane production, microbial protein synthesis and concentrations of protozoa, ammonia and VFA were determined as previously described (Belanche *et al.*^2013^). Concentrations of total lactate and D-Lactate were measured using the Enzytec D/L-Lactic Acid kit (r-biopharm, Darmstadt, Germany) while L-lactate was calculated as the difference between them. Enzymatic activities in vessels content were measured following the procedure described by Giraldo *et al*. (2008). Briefly, freeze-dried samples (200 mg) were diluted with phosphate buffer (1.6 mL) and bead-beaten for 3 min to release intracellular enzymes. Then, cell material was removed by centrifugation (10 000 × *g*; 10 min, 4 ºC), and the supernatant was used to determine the enzymatic activity and the protein content using the Bradford Protein Assay Procedure (Bradford Reagent, Sigma, USA). Carboxymethylcellulase (EC 3.2.1.4.), xylanase (EC 3.2.1.8.) and amylase activities (EC 3.2.1.1.) were determined using carboxymethylcellulose, oat beachwood xylan and soluble starch as substrates, respectively. Buffer (750 μL) was mixed with each substrate (250 μL of 10 g L^−1^) and incubated at 39ºC for 10 min at pH 6.5. Sample extract (100 μl) was added and incubated for a further 15 min at 39ºC. Di-nitrilo salicylic acid (1.5 mL) was added to each tube and placed in a boiling water bath for 5 min to stop the reaction. For all enzymatic activities, the absorbance was read at 540 nm using a spectrophotometer (BioTek, Potton, UK) against glucose or xylose standards (from 0 to 2 g L^−1^). Blanks containing only buffer, buffer plus substrate and buffer plus enzyme were also incubated to correct for substrate autolysis and sugars present in the enzyme fraction. Enzymatic activities were measured in triplicate and expressed as mmol of sugar released from the corresponding substrates in 1 min per gram DM of sample (or gram of protein).

### DNA extraction and quantitative PCR

Genomic DNA was extracted from vessel samples withdrawn at different time points. Lyophilized samples (100 mg DM) were bead-beaten for 1 min and DNA was extracted using a QIAamp DNA Stool Mini Kit (Qiagen Ltd, Crawley, UK) according to the manufacturer's instructions, but with the incubation temperature increased to 95ºC for 10 min to maximize microbial lysis. Concentration and quality of genomic DNA was assessed by spectrophotometry (Nanodrop ND-100, Thermo Scientific, USA).

Absolute concentrations of DNA from total bacteria, protozoa, anaerobic fungi and methanogens were determined by qPCR and serial dilutions of their respective standards (10^−1^–10^−5^) as previously explained (Belanche *et al.*^2012^). Briefly, quantitative PCR (qPCR) was conducted in triplicate using a LightCycler® 480 System (Roche, Mannheim, Germany). Samples were prepared in 384-well plates using Epimotion 5075 Liquid Handeling System (Ependorf, Stevenage, UK). Amplification reaction (12.5 μL) contained DNA template (1 μL), 1mM of each primer and 6.25 μl of SYBR Green JumpStart Taq ReadyMix (Sigma-Aldrich Ltd, Dorset, UK). Amplification conditions were 95ºC for 5 min, then 60 cycles at annealing temperatures described in Table S2, (Supporting Information) for 30 s, 72ºC for 30 s and 95ºC for 15 s, and a final melting analysis was performed to check primer specificity.

### Ion Torrent Next Generation Sequencing

Rumen bacteria and methanogenic archaea communities were studied using Next Generation Sequencing (NGS) (de la Fuente *et al.*^2014^). To improve sequencing depth, only one sample per vessel was sequenced containing pooled DNA from the different time points. For bacterial profiling, amplification of the V1–V2 hypervariable regions of the 16S rRNA was carried out using bacterial primers (27F and 357R) followed by Ion Torrent adaptors (Table S2, Supporting Information). For methanogens profiling, amplification of the V2-V3 hypervariable region of the 16S rRNA was performed using archaeal primers (86F and 519R) also followed by adaptors (Table S2, Supporting Information). Forward primers were barcoded with 10 nucleotides to allow the sample identification. PCR was conducted in duplicate; a 25 μl reaction was prepared containing DNA template (1 μl), 0.2 μM of each primer and 12.5 μL of KAPA HiFi Mix (Kapa Biosystems Ltd., London, UK). Amplification conditions for bacteria and methanogens were 95ºC for 3 min, then 25 cycles of 98ºC for 30 s, 58ºC for 30 s, 68ºC for 45 s with a final extension at 68ºC for 7 min. Resultant amplicons were visualized on a 1% agarose gel to assess quality of amplifications before pooling the duplicate reactions. PCR products were further purified using Agencout AMpure XP beads (Beckman Coulter Inc., Fullerton, USA), and DNA concentration was assessed using an Epoch Microplate Spectrophotometer fitted with a Take 3 Micro-Volume plate (BioTek, Potton, UK) to enable equimolar pooling of samples with unique barcodes. Libraries were further purified using the E-Gel System with 2% agarose gel (Life Technologies Ltd, Paisley, UK). Purified libraries were assessed for quality and quantified on an Agilent 21000 Bioanalyzer with High Sensitivity DNA chip (Agilent Technologies Ltd., Stockport, UK). The emulsion PCRs were carried out using the Ion PGM Template OT2 200 Kit, and the sequencing using the Ion Torrent Personal Genome Machine (PGM) system using the Ion PGM Sequencing 316 Chip v2 (Life Technologies Ltd, Paisley, UK). Due to the lower abundance of methanogens than total bacteria, methanogens library was sequenced using a smaller chip (Ion PGM Sequencing 314 Chip v2).

Following sequencing, data was processed as previously described (de la Fuente *et al.*^2014^). Briefly, sample identification numbers were assigned to multiplexed reads using the MOTHUR software environment. Data were denoised by removing low-quality sequences, sequencing errors and chimeras (quality parameters: maximum 10 homopolymers, Q15 average over a 50bp window, no mismatches allowed with barcode and one maximum with primer; Chimera check, both denovo and database driven using Uchime). Then, sequences were cluster into OTUS's at 97% identity using CD-HIT-OTU pipeline (http://eeizhong-lab.ucsd.edu/cd-hit-otu). The number of reads per sample was normalized to the sample with the lowest number of sequences. Bacterial taxonomic information on 16S rRNA transcripts was obtained by comparing against the Ribosomal Database Project-II, while the methanogens were compared with the RIM-DB database (Seedorf *et al.*^2014^). To exclude potential bacterial sequences from the methanogens dataset, methanogens sequences were blasted with the Ribosomal Database Project-II, and those annotations which matched with bacterial sequences were removed. Only annotations with a bootstrap value over 80% were assigned; otherwise, they were considered as unclassified. Raw sequences reads from the bacterial and methanogens libraries were deposited at the EBI Short Read Archive from the European Nucleotide Archive (accession number PRJEB9813 and PRJEB9814, respectively).

### Calculations and statistical analyses

Microbial-N contribution to overflow was estimated based on the relationship between the ^15^N enrichment in the total digesta (NAN) and in the total bacteria pellet (Microbial-N: NAN = Digesta NAN ^15^N enrichment: total bacteria ^15^N enrichment). The ability of bacteria to incorporate ammonia was calculated as the ratio between their ^15^N enrichments (Total bacteria ^15^N enrichment: ammonia ^15^N enrichment). For diet disappearance, N metabolism and microbial diversity data were analysed using an ANOVA (Genstat 15th edition, VSN International, UK) as follow.

\[
{Y_{ijk}} = \mu + {D_i} + {A_j} + {e_{ijk}},
\]

where *Y_ijk_* is the dependent, continuous variable (*n* = 4), *μ* is the overall mean; *D_i_* is the fixed effect of the diet (*i* = CON, CHI, IVY), *A_j_* is the random effect of the animal inoculum (*j* = 1 to 4) and *e_ijk_* is the residual error. For the rumen fermentation and qPCR data was analysed using a repeated-measurements procedure (REML) including the different time-points after feeding (2, 4, 8 and 24 h). When significant effects were detected, treatment means were compared by Fisher's protected LSD-test. Findings with *P* < 0.05 were regarded statistically significant, while *P* < 0.1 was considered as a tendency to differences.

Dietary effects on NGS log-transformed data were analysed based on their Bray–Curtis distance metric within the function UPGMA. Data were then analysed by non-parametric permutational multivariate analysis of variance using PRIMER-6 software (PRIMER-E Ltd., Plymouth, UK). Pairwise comparisons were also conducted to elucidate differences between treatments. The pseudo-*F* statistics and *P*-values were calculated after 999 random permutations of residuals under a reduced model using the Monte Carlo test. A canonical correspondence analysis (CCA) was also performed to investigate the relationships between the structure of the bacterial and methanogens communities and the fermentation pattern. The signification of each variable was calculated using 999 random permutations. Bacterial and methanogens biodiversity indexes were calculated using normalized data to reduce overinflation of true diversity in pyrosequencing data sets. For bacterial and methanogens relative abundances, data were tested for normality and homogeneity using the Shapiro–Wilk and the Bartlett's tests, respectively. Then, data were log transformed and *P-*values were adjusted for multiple testing using the method proposed by Benjamini and Hochberg (1995) to decrease the False Discovery Rate.

## RESULTS

### Fermentation pattern

Inclusion of 5% of CHI or IVY in diets for ruminants did not have any detrimental effects on feed disappearance after 48 h-incubation in the Rusitec system (Table 1). IVY promoted a strong decrease in gas production and a moderate decrease in methane concentration, while the opposite was true for CHI. As a result, both feed additives caused a similar decrease in methane emissions. Theoretical metabolic hydrogen production based on the VFA stoichiometry tended to decrease more for CHI than for IVY in comparison to CON diet.

**Table 1.** Effect of supplementing a control diet (CON) with chitosan (CHI) or ivy fruit saponins (IVY) on feed disappearance and methanogenesis in the Rusitec system.

Diets^1^	CON	CHI	IVY	SED	*P-*value
**Disappearance (%)**					
OM	68.6	68.6	72.5	1.99	0.157
N	60.0	61.6	63.9	2.99	0.463
C	65.4	65.0	69.9	2.12	0.108
NDF	41.9	41.5	42.8	2.67	0.881
ADF	37.1	33.0	39.7	3.29	0.201
**Gas emissions**					
Total gas (L d^−1^)	2.69^a^	2.47^ab^	2.16^b^	0.127	0.016
Methane (mM)	1.94^a^	1.22^c^	1.50^b^	0.079	<0.001
Methane (mmol d^−1^)	5.24^a^	2.99^b^	3.26^b^	0.289	<0.001
Methane (mmol gDOM^−1^)	0.40^a^	0.22^b^	0.23^b^	0.018	<0.001
[H] produced^2^ (mmol d^−1^)	14.5	10.4	12.5	1.43	0.075

1 Diets with 5% inclusion rate (*n* = 4). a,b,c: Within a row means without a common superscript differ (*P* < 0.05).

2 Metabolic hydrogen production stoichiometrically calculated based on VFA production (Moss, Jouany and Newbold 2000).

Diurnal changes in the fermentation pattern (Table 2) as a result of the feeding regimen used in Rusitec (one feeding per day) were observed. Total concentration of VFA progressively increased from feeding to 8 h after-feeding, and this was accompanied by a decrease in pH and ammonia concentration. Samples taken 2 h after feeding had the highest concentrations of total lactate, L-lactate and acetate molar proportion (*P* < 0.001), as well as the lowest proportions of propionate and butyrate. In terms of absolute enzymatic activity (Table 3), the peak of fermentation occurred at 2 h after feeding for amylase and CMC-ase, but no diurnal differences were observed in the relative enzymatic activity.

**Table 2.** Effect of supplementing a control diet (CON) with chitosan (CHI) or ivy fruit saponins (IVY) on rumen fermentation parameters in the Rusitec system.

	Diets^1^				*P-*value	Time after feeding					*P-*value	
	CON	CHI	IVY	SED	Diet	2 h	4 h	8 h	24 h	SED	Time	Interaction
pH	5.94	5.87	6.00	0.044	0.054	5.95^b^	5.79^c^	5.76^c^	6.24^a^	0.013	<0.001	<0.001
Ammonia-N (mg L^−1^)	1.44	2.18	0.56	0.509	0.051	1.93^a^	0.76^b^	0.49^b^	2.39^a^	0.310	0.001	0.085
Total VFA (mM)	135	130	130	3.96	0.341	125^c^	140^b^	145^a^	116^d^	2.390	<0.001	0.364
Molar proportion												
Acetate	54.1^a^	48.5^b^	49.4^b^	0.96	0.002	51.9^a^	51.2^ab^	50.8^b^	48.9^c^	0.331	<0.001	0.037
Propionate	24.7^b^	33.8^a^	25.3^b^	2.59	0.022	26.8^c^	27.5^b^	27.9^b^	29.5^a^	0.274	<0.001	0.035
Butyrate	10.3^a^	6.76^b^	11.9^a^	0.93	0.004	9.49^b^	9.54^b^	9.68^ab^	9.91^a^	0.127	0.027	0.055
Iso-butyrate	0.73^a^	0.41^b^	0.78^a^	0.031	<0.001	0.62^b^	0.59^b^	0.62^b^	0.73^a^	0.020	<0.001	0.149
Valerate	4.66^c^	7.18^a^	5.79^b^	0.281	<0.001	5.82^bc^	6.07^a^	5.89^b^	5.73^c^	0.077	0.002	0.205
Iso-valerate	1.64^a^	0.57^b^	1.82^a^	0.292	0.011	1.22	1.26	1.32	1.58	0.118	0.073	0.236
Lactate (mM)												
Total	1.61^b^	2.47^a^	1.98^ab^	0.214	0.020	3.81^a^	1.62^b^	0.84^c^	1.80^b^	0.297	<0.001	0.038
D-lactate	0.83^b^	1.39^a^	0.86^b^	0.133	0.010	1.49^a^	0.69^b^	0.37^b^	1.56^a^	0.179	<0.001	0.096
L-lactate	0.78	1.08	1.12	0.140	0.096	2.32^a^	0.94^b^	0.47^c^	0.23^c^	0.177	<0.001	0.05
Ratio D/L	2.00	3.66	1.37	0.866	0.089	0.66^b^	0.81^b^	0.76^b^	7.14^a^	1.032	0.002	0.124

1 Diets with 5% inclusion rate (*n* = 4). a,b,c: Within a row means without a common superscript differ (*P* < 0.05).

**Table 3.** Effect of supplementing a control diet (CON) with chitosan (CHI) or ivy fruit saponins (IVY) on rumen enzymatic activity and microbial numbers in the Rusitec system.

	Diets^1^				*P-*value	Time after feeding					*P-*value	
	CON	CHI	IVY	SED	Diet	2 h	4 h	8 h	24 h	SED	Time	Interaction
Absolute enzymatic activity^2^												
Amylase	0.95^b^	2.43^a^	1.00^b^	0.445	0.026	1.95^a^	1.62^ab^	1.41^b^	0.86^c^	0.229	0.001	0.354
Xylanase	0.26	0.26	0.30	0.042	0.588	0.29	0.26	0.24	0.29	0.024	0.173	0.117
Carboxymetyl-cellulase	0.09	0.08	0.08	0.017	0.910	0.10^a^	0.09^a, b^	0.07^b^	0.08^b^	0.010	0.027	0.037
Relative enzymatic activity^2^												
Amylase	2.03	5.27	2.75	1.441	0.139	4.06	3.84	3.47	2.03	0.741	0.099	0.306
Xylanase	0.64	0.56	0.72	0.105	0.387	0.63	0.63	0.67	0.62	0.086	0.821	0.307
Carboxymetyl-cellulase	0.22	0.19	0.20	0.053	0.925	0.22	0.21	0.19	0.20	0.047	0.755	0.215
Microbial numbers^3^												
Bacteria (pg gDM^−1^)	9.18	9.28	9.03	0.108	0.142	8.95^b^	9.21^a^	9.29^a^	9.21^a^	0.092	0.012	0.876
Methanogens (copies gDM^−1^)	9.94	9.74	9.68	0.165	0.323	9.57^b^	9.91^a^	9.76^a, b^	9.91^a^	0.115	0.028	0.170
Methanogens (10^3^×ΔC_T_)	3.27^a^	1.78^b^	2.44^ab^	0.467	0.049	2.56	2.54	1.70	3.18	0.727	0.275	0.201
Anaerobic fungi (10^8^×pg gDM^−1^)	2.12^a^	3.22^a^	–1.91^b^	1.218	0.013	1.14	2.70	0.09	0.63	1.379	0.289	0.610
Protozoa (pg gDM^−1^)	4.14	3.29	3.32	0.561	0.301	3.19	3.38	4.02	3.75	0.346	0.132	0.405
Protozoa (cells mL^−1^)	2.41	2.00	1.88	0.273	0.206							

1 Diets with 5% inclusion rate (*n* = 4). a,b,c: Within a row means without a common superscript differ (*P* < 0.05).

2 Absolute and relative enzymatic activity were expressed in: (μmol of sugar gDM^−1^ min^−1^) and (μmol of sugar g^−1^ Protein min^−1^), respectively.

3 Microbial numbers were log-transformed to attain normality. Protozoal samples were taken before feeding and measures by optical microscopy.

**Figure 1.**
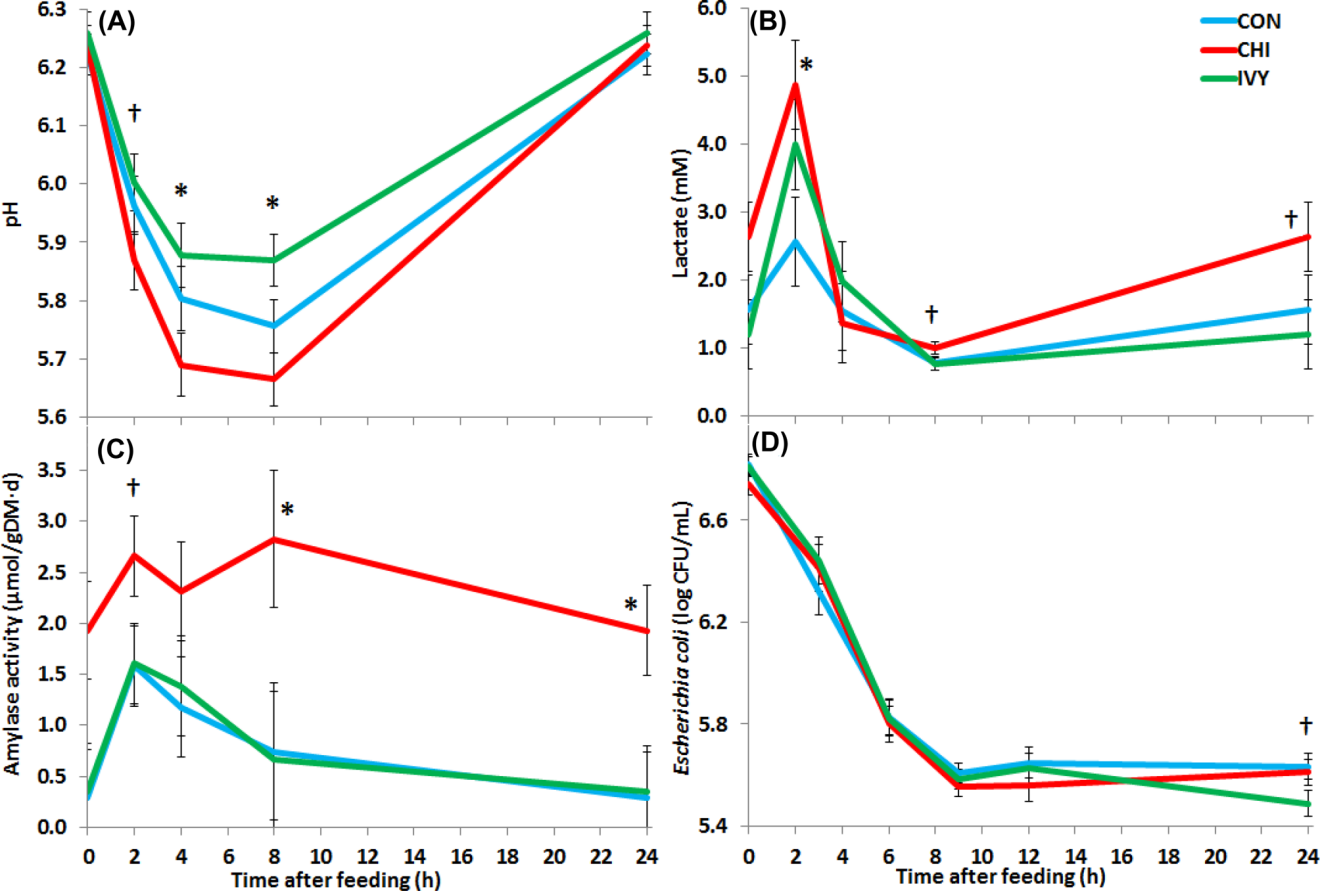
Effect of supplementing a control diet (CON) with chitosan (CHI) or ivy fruit saponins (IVY) on pH (**A**), total lactate concentration (**B**), amylase activity (**C**) and *E. coli* concentrations post-inoculation (**D**) in the Rusitec system. Error bars indicate the standard error of the difference for each time point (*n* = 4). *P < 0.05, ^†^*P* < 0.1.

All experimental diets led to similar diurnal changes in most of the fermentation parameters consisting of a progressive decrease in pH and concentrations of ammonia and lactate after feeding. This was accompanied by a concomitant increase in total VFA concentration which peaked at 8 h after feeding for all treatments. Thus, only the main factors (Diet and Time) are described in Tables 2 and 3. Those parameters which had significant interactions (Diet × Time) were further investigated to explore the potential effect of CHI and IVY on synchronization between energy and N availability for the rumen microbes (Fig. 1). Total VFA concentration in the fermentation vessels was unaffected by the experimental diets, but modified the molar proportion of the main VFA (Table 2). Both CHI and IVY decreased acetate and increased valerate molar proportions. Moreover, CHI promoted an increase in the proportion of propionate, and decreased the proportions of butyrate, isobutyrate and isovalerate. Inclusion of CHI in the diet tended to increase ammonia concentration, particularly at 2 h after feeding (*P* = 0.007). CHI also increased total and D-lactate concentrations, with the highest differences observed 2 h after feeding (*P* < 0.05). Moreover, CHI boosted fermentation late after feedings as there was a tendency towards increase total lactate concentration (*P* < 0.10) and amylase activity (*P* < 0.05) after 8 post-feeding. On the contrary, IVY tended to promote the lowest ammonia concentration across all-time points and buffered the post-prandial drop in rumen pH, with the greatest differences recorded at 4 and 8 h after feeding (*P* < 0.05). No further differences across diets were observed for the absolute xylanase and CMC-ase activity (μmol of sugar released gDM^−1^ min^−1^) nor for the relative enzymatic activity (μmol of sugar released g Protein^−1^·min^−1^).

Quantitative PCR revealed diurnal changes in the absolute concentration of bacterial and methanogen DNA, with the lowest values observed at 2 h after feeding, but no differences across diets were noted. In comparison to CON diet, IVY decreased the anaerobic fungi concentration whilst CHI decreased the relative abundance of methanogens to total bacteria. Protozoal concentration in the fermenters remained low (below 500 cells mL^−1^) and unaffected by the experimental treatments. This protozoal population was mainly composed of subfamily Entodiniinae 96.1±4.1%, followed by Diplodiniinae 3.5±4.2% and Holotrichs 0.4±0.7%, and these proportions were unaffected by the treatments.

### Bacterial 16S rDNA sequencing

**Figure 2.**
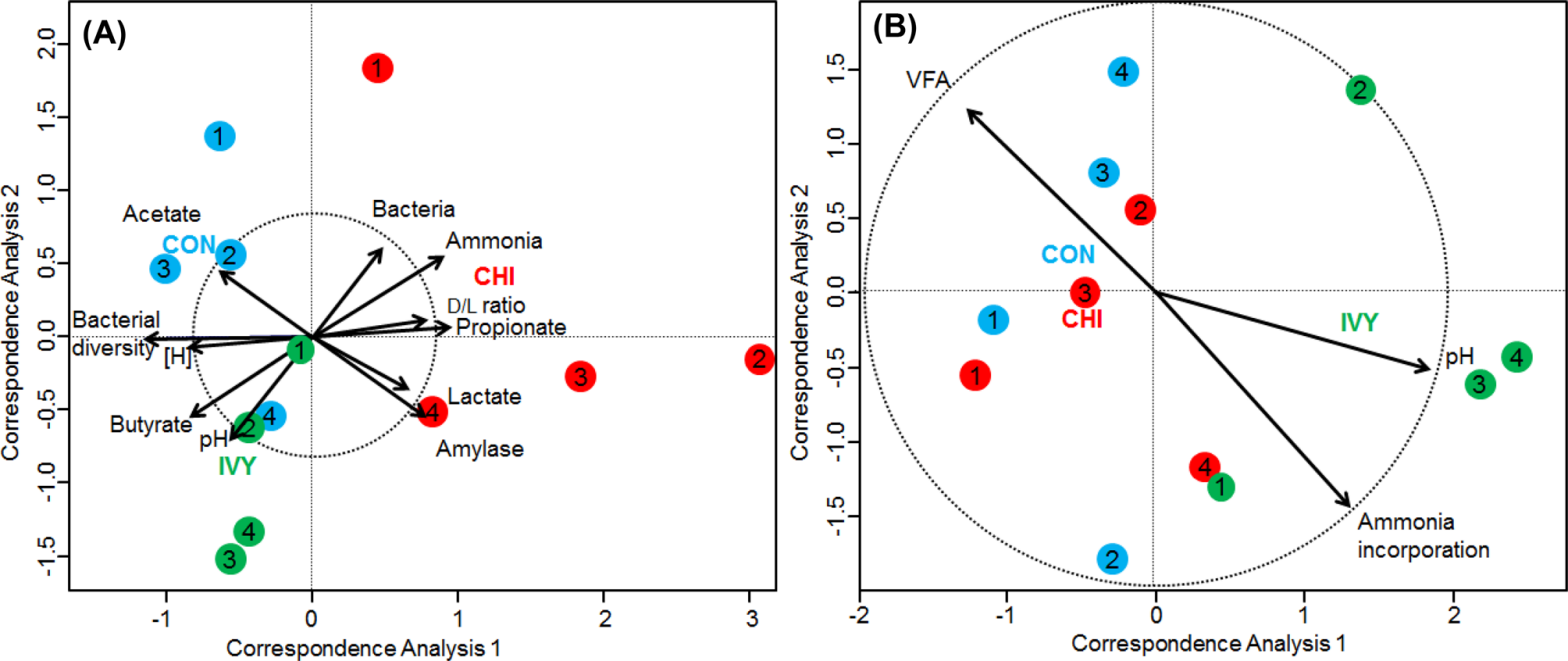
CCA illustrating the relationship between the structure of the bacterial community (**A**) or methanogen community (**B**) with the rumen fermentation pattern in the Rusitec system. Arrows show the direction of the gradient and their length is proportional to the correlation. Arrows longer that the dotted circle are significant (*P* < 0.05). Centroid is indicated for each treatment: Control (CON), chitosan (CHI) and ivy fruit saponins (IVY). Animals used as donors are indicated in numbers.

Bacterial 16S rDNA sequencing generated 2.1 million of raw sequences. Quality filtering resulted in 587 792 high-quality sequences (average length of 285 bp) that were clustered in to 833 unique OTUs with 29 679 sequences per sample after normalization. Permutational analysis of variance (Table 4) demonstrated a strong effect of the experimental diets on the structure of the bacterial community. Pair-wise analysis showed that the structure of the bacterial community in CHI diets differed to that observed in CON and IVY, while no differences were observed between CON and IVY. These differences were particularly obvious within the phylum Proteobacteria (Fig. S1, Supporting Information). In order to detect possible correlations between the structure of the bacterial community (samples) and the rumen fermentation, a CCA was performed. Fig. 2A shows a clear separation of the CHI samples on the ordination plot in respect to those from CON and IVY (horizontal axis). Moreover, several variables were correlated, or tended to be correlated, with this sample distribution: vessel concentration of ammonia (*P* = 0.004); propionate (*P* = 0.025); total bacteria (*P* = 0.082); lactate (*P* = 0.090); and D/L ratio (*P* = 0.082), as well as amylase activity (*P* = 0.011) were positively correlated to the structure of the bacterial community of CHI samples, whereas bacterial diversity (*P* = 0.001) and metabolic hydrogen production (*P* = 0.043) were negatively correlated. Concentration of butyrate (*P* = 0.004) and pH (*P* = 0.018) were positively correlated to IVY samples, while acetate concentration tended to be correlated to CON samples (*P* = 0.077).

**Table 4.** Effect of supplementing a control diet (CON) with chitosan (CHI) or ivy fruit saponins (IVY) on the structure of the bacterial and methanogen communities in the Rusitec system.

	Bacteria			Methanogens		
Community	Similarity	Pseudo- *F*	*P-*value	Similarity	Pseudo- *F*	*P-*value
Treatment effect		4.54	0.007		9.06	0.003
Pairwise comparisons						
CON versus CHI	59.5	2.33	0.046	76.0	1.09	0.360
CON versus IVY	73.9	1.62	0.118	62.4	3.10	0.024
CHI versus IVY	60.1	2.17	0.048	60.7	7.00	0.003

Microbial communities were studied using NGS. Permutational analysis of variance was performed using Bray–Curtis similarity measurements of log-transformed number of sequences: higher Pseudo-*F* and lower similarities and *P-*values correspond to greater differences in the microbial composition. Diets with 5% inclusion rate (*n* = 4).

**Figure 3.**
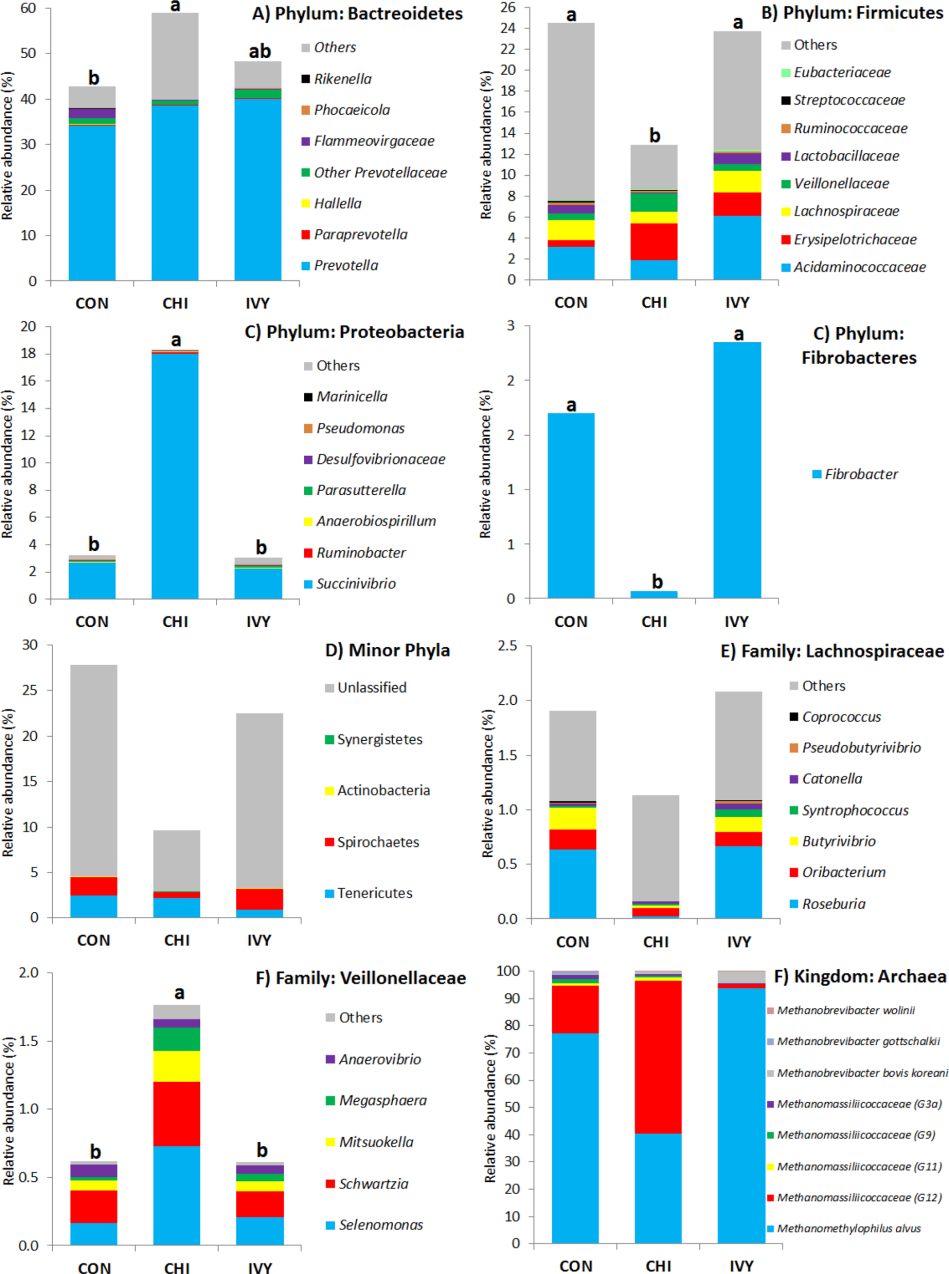
Effect of supplementing a control diet (CON) with chitosan (CHI) or ivy fruit saponins (IVY) on abundance of the main bacteria (**A**, **B**, **C**, **D**, **E** and **F**) and archaea (F) phyla and families in the Rusitec system. a,b: Within a row means without a common superscript differ (*P* < 0.05).

In terms of bacterial diversity (Table 5), no differences across diets were observed for Chao index and Good's coverage, indicating that the sequencing depth was comparable across treatments. CHI decreased Shannon and Simpson indexes indicating a decreased bacterial diversity. Moreover, the lower Evenness values in CHI diets indicated the presence of highly abundant species together with minor species, while in CON and IVY there was a greater similarity in the abundance across bacterial species. Based on the classification by RDPII (Fig. 3), Bacteroidetes was the most abundant phylum across diets (50%), followed by Firmicutes (20%), Proteobacteria (8.2%), Tenericutes (1.8%), Spirochaetes (1.7%), Fibrobacteres (1.4%), minor phyla (0.1%), whereas some sequences were unclassified (16%). Differences between diets were observed at the phylum level (Fig. S1; Table S3, Supporting Information). CHI increased the abundance of Bacteroidetes and Proteobacteria and decreased Firmicutes, Spirochaetes, Fibrobacteres and unclassified bacteria. CHI also promoted a shift at the family level within the Firmicutes phyla, increasing the abundance Veillonellaceae, family in which the genera *Megasphaera, Mitsuokella, Schwartzia* and *Selenomonas* are included. On the contrary, inclusion of IVY in the diet did not modify the numbers of most of the bacteria studied and only increased the abundance of Tenericutes in respect to CON. CHI and IVY had no specific antimicrobial effect against *Escherichia coli* (Fig. 1D) since post-inoculation concentration of the pathogen in the vessels followed a similar decay pattern with all three diets.

**Table 5.** Effect of supplementing a control diet (CON) with chitosan (CHI) or ivy fruit saponins (IVY) on the structure of the bacterial and methanogen communities in the Rusitec system.

Diets^1^	CON	CHI	IVY	SED	*P-*value
**Bacteria**					
Richness	578	433	521	62.8	0.146
Shannon	4.75^a^	4.09^b^	4.74^a^	0.178	0.015
Evenness	0.75^a^	0.68^b^	0.76^a^	0.015	0.003
Simpson	0.98^a^	0.95^b^	0.98^a^	0.009	0.033
Chao	708	519	696	111.1	0.243
Good's	0.82	0.80	0.79	0.030	0.602
**Methanogens**					
Richness	8.50	8.75	5.00	1.550	0.091
Shannon	0.64^a^	0.64^a^	0.26^b^	0.128	0.041
Evenness	0.31	0.29	0.19	0.071	0.276
Simpson	0.32	0.33	0.12	0.100	0.135
Chao	9.00	9.13	5.38	1.71	0.133
Good's	0.84	0.82	0.77	0.098	0.784

1 Diets with 5% inclusion rate (*n* = 4). a,b: Within a row means without a common superscript differ (*P* < 0.05).

### Methanogens 16S rDNA sequencing

Methanogen sequencing generated 0.29 million raw sequences. Quality filtering resulted in 70 298 high-quality sequences (average length of 380 bp) that were clustered in to 11 unique OTU's with 1167 sequences per sample after normalization. Permutational analysis of variance revealed a strong effect of diet on the structure of the methanogen community. Pair-wise analysis showed that IVY promoted a methanogen community with a structure which differed to that observed in CON and CHI, while no differences were observed among CON and IVY. Similarly, CCA showed a clear separation between IVY samples and those from CON and CHI according to the horizontal axis in the ordination plot (Fig. 2B). However, the structure of the methanogen community was not correlated with most of the fermentation parameters; only pH (*P* = 0.066) and ammonia incorporation (*P* = 0.056) tended to be positively correlated, while total VFA concentration tended to be negatively correlated (*P* = 0.095) with the structure of the methanogen community of IVY samples.

Similarly to bacteria, methanogen 16S rDNA sequencing showed no differences across diets on Chao index and Good's coverage, indicating a homogeneous sequencing depth across treatments (Table 5). IVY tended to decrease the methanogens diversity in terms of richness and Shannon index, while no differences were observed between CON and CHI. Evenness was low and constant across dietary treatments indicating that the methanogens population is dominated by the presence of major species with less contribution from minor species. Based on the RIM-DB database (Fig. 3), only two families made up the entire methanogen population: Methanomassiliicoccaceae (class Thermoplasmata) and Methanobacteriaceae (class Methanobacteria; Fig. S2, Supporting Information; Table 4). IVY tended to decrease the abundance of the family Methanomassiliicoccaceae, promoting a substitution of Group 12 by Group 11 (i.e. *Methanomethylophilus albus*). On the contrary, CHI promoted a substitution of Group 11 by Group 12.

### Fermentation products and microbial protein synthesis

Similar daily yields of total VFA and DM were observed across diets (Table 6); however, the composition of these fermentation products differed among diets. CHI increased propionate outflow in detriment to butyrate. CHI also increased the outflow of ammonia-N and microbial-N, as well as the efficiency of microbial protein synthesis. On the contrary, IVY decreased the outflow of ammonia-N, non-ammonia-N and microbial-N. As a result, IVY trended to have a negative impact on the efficiency of microbial protein synthesis in terms of microbial-N per unit of N intake, degradable N and degradable OM. Ammonia represented almost the only N source for microbes fed IVY diets (99%) but not for those fed CHI diets (63%).

**Table 6.** Effect of supplementing a control diet (CON) with chitosan (CHI) or ivy fruit saponins (IVY) on fermentation products and microbial protein synthesis in the Rusitec system.

Diets^1^	CON	CHI	IVY	SED	*P-*value
**Fermentation products (mmol d^−^^1^**)					
Total VFA	63.3	57.8	60.0	4.79	0.549
Acetate	34.1	30.1	30.7	2.96	0.403
Propionate	13.0	16.5	13.8	1.20	0.059
Butyrate	6.81^a^	3.44^b^	6.22^a^	0.594	0.003
Isobutyrate	0.35^a^	0.26^a^	0.03^b^	0.030	0.021
Valerate	4.00	4.85	4.27	0.517	0.317
Isovalerate	1.60	0.81	1.38	0.477	0.305
Caproate	2.89^a^	1.37^b^	2.72^a^	0.435	0.025
CH_4_:VFA (mol:mol)	0.087^a^	0.055^b^	0.057^b^	0.0036	<0.001
**^15^N enrichment**					
Ammonia	0.89	0.88	0.79	0.085	0.481
Bacteria	0.69^a^	0.54^b^	0.77^a^	0.041	0.004
Digesta	0.34^a^	0.28^b^	0.39^a^	0.021	0.006
**Outflows (g d^−^^1^)**					
DM	22.3	22.2	21.4	0.40	0.147
Ammonia-N	0.012^b^	0.026^a^	0.004^b^	0.0033	0.002
NAN	0.46^a^	0.49^a^	0.40^b^	0.016	0.004
NANM-N^2^	0.23^a^	0.23^a^	0.20^b^	0.010	0.031
Microbial-N	0.23^b^	0.26^a^	0.20^c^	0.008	0.001
**Efficiency of synthesis (g g^−^^1^)**					
Microbial-N : NAN	0.50	0.53	0.51	0.023	0.229
Microbial-N : N intake	0.55	0.56	0.51	0.019	0.095
Microbial-N : Degradable N	0.91	0.91	0.80	0.045	0.079
Microbial-N from ammonia	0.79	0.63	0.99	0.114	0.053
Microbial-N : DOM (g kg^−1^)	17.2^b^	19.5^a^	14.6^c^	0.65	<0.001

1 Diets with 5% inclusion rate (*n* = 4). a,b,c: Within a row means without a common superscript differ (*P* < 0.05).

2 NANM-N; non-ammonia non-microbial N calculated by subtracting microbial N from non-ammonia N-flow.

## DISCUSSION

### Rumen fermentation and methane emissions

Chitinase and chitosanase activity has been described in only a few rumen bacteria and protozoa (Morgavi *et al.*^1994^). In particular, two strains of *Clostridium tertium ChK5* have been identified as the most active bacteria able to degrade chitosan in the rumen (Kopecny and Hodrova 2000). However, in our experiment low and constant numbers for protozoa (average 125 cells mL^−1^) and *C. sensu stricto*, genus to which *C. tertium* belongs (0.05% of the total bacteria), were observed across treatments. Therefore, it seems unlikely that chitosan was directly degraded due to chitosanase activity ‘per se’. Despite this, many commercial enzymes with different original specificities, such as cellulase, pectinase (Kittur, Kumar and Tharanathan 2003), pepsin (Roncal *et al.*^2007^), neutral protease (Li *et al.*^2005^) and lipase (Lee, Xia and Zhang 2008) have been reported for their abilities to hydrolase chitosan. Moreover, it has been observed that incubation of chitosan with a commercial α-amylase (optimal conditions: 160 IU/mL, pH 5 and 50ºC for 4 h) was able to hydrolase the β-(1–4) glycosylic bonds in chitosan producing chitooligosaccharides as end products (Wu 2011). Although the fermentation conditions in our experiment differed to those described above, it is likely that some degradation of chitosan occurred. The increase in amylase activity (2.6-fold times) observed in vessels fed CHI in comparison to those fed CON seems to support this hypothesis. These chitooligosaccharides can further be used by some gut bacteria as carbon source (Chen *et al.*^2002^) and could explain to some extent the change in the bacterial community and ultimately the greater production of propionate (+27%) and lactate (+53%) as fermentation products. These results are in line with recent publications which reported linear increments in propionate production *in vivo* and *in vitro* as a response of chitosan incorporation into the diet (Araujo *et al.*^2015^; Belanche, Ramos-Morales and Newbold 2015).

Additionally, chitosan and chitooligosaccharides have also been described to have important antimicrobial properties (Kong *et al.*^2010^). Due to these antimicrobial properties, excessive doses of chitosan are generally not recommended in order to prevent negative effects on rumen function (Goiri, Garcia-Rodriguez and Oregui 2009b). In a similar experiment to ours, a substantial reduction in methane emissions (–42%), together with decreased feed digestibility was reported when high soluble chitosan (95% deacetylated) was used in a Rusitec systems at 7% inclusion rate into the diet (Goiri, Garcia-Rodriguez and Oregui 2009b). Our data showed that similar reductions in methane production (–43%) without a negative impact on feed digestibility can be achieved by decreasing slightly chitosan solubility (85% deacetylated) and its inclusion rate (5%) in mixed diets. This finding is in line with recent publications, which reported no negative impact of chitosan on DM digestibility when low-forage diets are used *in vivo* (Goiri, Oregui and Garcia-Rodriguez 2010; Araujo *et al.*^2015^) or *in vitro* (Wencelova *et al.*^2014^).

The addition of IVY to the diet promoted a similar decrease in rumen methanogenesis (–40%) to that observed for CHI with no effect on feed digestibility. In a meta-analysis compiling information from 16 experiments in which different types of saponins were used (Patra 2010), it was concluded that on average saponins trend to decrease methane emissions (–11%) by decreasing rumen protozoa concentration (–28%) and NDF digestibility (–11%); however, these effects are often inconsistent and highly affected by the type of saponins considered (Patra and Saxena 2009a). In a preliminary study in which rumen protozoa and ^14^C-labelled bacteria were incubated *in vitro* (Belanche, Ramos-Morales and Newbold 2015), we demonstrated that both CHI and IVY have a strong antiprotozoal effect when used at concentrations above 1g L^−1^. In the present study, the antiprotozoal effect of the feed additives was not observed, possibly due to the inherent difficulty of maintaining high numbers of protozoa in Rusitec (Belanche *et al.*^2013^). Thus, the antiprotozoal effects of CHI and IVY need to be further studied *in vivo* to elucidate its mode of action and its duration in time.

CHI promoted an increase in lactate concentration (+53%), which together with the increase in the Genus *Megasphaera* (+0.80 log units) and other lactate utilizers such as *Selenomonas* (+0.66) and *Veillonella* (+0.46), could explain to some extent the increased propionate production (+27%) as fermentation product. Thus, CHI did not modify total VFA production but substantially shifted the fermentation pattern from acetate towards propionate production, tending to decrease (–28%) theoretical metabolic hydrogen production (Moss, Jouany and Newbold 2000). This shift in fermentation was, therefore, the main antimethanogenic driver for CHI explaining two thirds of the observed decreased in methanogenesis. As a result, CHI promoted a strong decrease in the CH_4_:VFA ratio (–34%) indicating that more energy was captured in fermentation products. Araujo *et al*. (2015) reported that increasing inclusion of chitosan into steers diet (up to 150 mg/g body weight) promoted a linear increase in rumen propionate proportion, blood glucose and feed digestibility without having a negative impact on DM intake. However, since it has been reported that intraruminal infusion of propionate can have a negative impact on feed intake and milk fat concentration (Sheperd and Combs 1998; Oba and Allen 2003), more studies are needed to rule out potential adverse effects of CHI when used a greater inclusion levels.

On the contrary, the addition of IVY to the diet modified neither total VFA production nor fermentation pattern. Thus, the predicted decrease in metabolic hydrogen production according to the fermentation products was very limited (–13%) and only explained one-third of the observed reduction in methane emissions. These observations agree with the literature which reported no differences in total VFA concentrations with supplementation of saponins, with the small decrease in the acetate to propionate ratio generally explained by the decrease in protozoal numbers (Patra and Saxena 2009a). Since IVY decreased methane emissions per gram of disappeared OM (–41%) without substantially changes in protozoal numbers and fermentation pattern, it seems to indicate that its main antimethanogenic mode of action under our experimental conditions are due to a direct inhibition of the methanogen comunity.

### Rumen bacterial community

Total numbers of bacteria, protozoa and methanogens were unaffected by the inclusion of CHI in the diet which, together with the similar VFA production and enzymatic activity, suggests that CHI does not have detrimental effects on the overall rumen fermentation. The main antimicrobial mode of action of chitosan polymers has been described to be based on a change in cell permeability due to interactions between the polycationic chitosan (R-NH_3_^+^), and the electronegative charges on the microbial surfaces when rumen pH is below the molecule's p*K*a (6.3–6.5) (Kong *et al.*^2010^). These electrostatic interactions promote hydrolysis of the peptidoglycans in the microorganism wall and ultimately cell lysis (Sudarshan, Hoover and Knorr 1992). Since the peptidoglycan layer is more accessible in Gram-positive than in Gram-negative bacteria, CHI antibacterial activity tended to cause a decline in the abundance of the former bacterial group in favour of the later one. As a result, CHI caused an important shift in the structure of the bacterial community promoting a less diverse community. Indeed, CHI decreased the abundance of *Firmicutes* (–0.33 log untis) and *Fibrobacter* (–1.28 log) but increased *Bacteroidetes* (+0.14 log) and *Proteobacteria* (+0.71 log), which include most of the amylolytic bacteria. This substitution of fibrolytic by amylolytic bacteria may explain the increased amylase activity detected in vessels fed CHI diets, and thus, the high abundance of propionate and lactate as fermentation products. Moreover, our CCA indicated that changes in the structure of the bacterial community induced by CHI were positively correlated with increasing concentrations of lactate, D/L ratio and low pH, suggesting that CHI activity is pH-dependent. Indeed, hydrophobic and chelating reactions have been described as the main antibacterial mode of action of CHI when pH is above its p*K*a (Kong *et al.*^2010^).

Recent publications have reported a negative impact of chitosan on total tract NDF digestibility in sheep fed chitosan at 136 mg kg^−1^ of BW (Goiri, Oregui and Garcia-Rodriguez 2010), as well as, on DM digestibility when forage-based diets are used *in vitro* (Wencelova *et al.*^2014^). The reason for that has been suggested to be the adverse effect of chitosan on rumen cellulolytic protozoa (Wencelova *et al.*^2014^; Belanche, Ramos-Morales and Newbold 2015). In this paper, we demonstrated that CHI has also a negative impact on the abundance of most of the rumen cellulolytic bacteria, such as *Fibrobacter* (–1.28 log units), *Butyrivibrio* (–0.90 log) and *Ruminococcus* (–0.28 log), and hemicellulolytic bacteria such as *Eubacterium* (–0.27 log). In our experiment, this decrease of fibrolytic bacteria had however no effect on CMC-ase activity and fibre disappearance. CCA showed that this shift in the bacterial community tended to be positively correlated with the concentration of total bacteria (+0.10 log units) and could compensate the lower abundance of fibrolytic bacteria. Therefore, the observed decrease in fibrolytic bacteria (most of which are Gram-positive) together with the increase in amylolytic bacteria (which are predominantly Gram-negative), and amylase activity seems to support the notion that CHI mode of action is based on an electrostatic interaction with the bacterial cell wall (Sudarshan, Hoover and Knorr 1992). Alternatively, the potential hydrolysis of CHI by amylases (Wu 2011) could also favour the proliferation of those bacteria able to use CHI as energy source (i.e. amylolytic bacteria) leading to changes in the structure of the bacterial community and on the fermentation products. Thus, more research is needed to elucidate which bacteria species are effectively able of utilizing CHI as energy source in the rumen.

The effect of saponins on the rumen concentrations of bacteria, fungi and methanogens is the balance between the direct antimicrobial effects of saponins (negative impact) and the indirect ant-protozoal effect (positive impact on the microbial growth) (Patra and Saxena 2009b). Low protozoal numbers were observed in our experiment across treatments, and thus prevented the antiprotozoal activity being accurately assessed, but allowed the effects of IVY on bacterial and methanogen communities to be investigated without a strong protozoal interference. Previous reports have indicated that this antiprotozoal is based on the sterol-binding capability of saponins to the protozoal cell membranes, but its effect on the bacterial community is still not clear (Patra and Saxena 2009b). Our results, however, showed no effect of IVY on total bacterial numbers, and CCA showed similar structure of the bacterial community in fermenters fed CON and IVY diets, as well as similar bacteria diversity, and genera distribution indicating that IVY had a limited antibacterial effect.

### Methanogen community

Methanogenic archaea are the sole producers of methane in the rumen (Morgavi *et al.*^2010^); therefore, a correlation between methanogens and methanogenesis might be expected (first hypothesis). However, a shift in the methanogen community towards a one less effective in producing methane might also been suggested to explain the differences in methane emissions (second hypothesis) (Hegarty 1999). This latter hypothesis is based on the different methanogenic potential observed among various methanogen groups (Hook, Wright and McBride 2010; Leahy *et al.*^2013^). This experiment demonstrated that CHI and IVY had different impacts on the methanogen population: CHI did not modify the structure and diversity of the methanogen community in comparison to CON but lowered the relative abundance of methanogens respect to total bacteria (–45%) and promoted a substitution of certain genera within the family *Methanomassiliicoccaceae*. A positive correlation, (*R* = 0.49–0.55), has recently been observed between the relative abundance of methanogens and methane emissions *in vivo* (Wallace *et al.*^2014^; Belanche, de la Fuente and Newbold 2015). Thus, according to the first hypothesis, the decrease in the methanogen numbers could explain one third the observed decreases in methanogenesis induced by CHI.

On the contrary, IVY did not modify the abundance of methanogens in the vessels, but promoted a profound change in the structure of the methanogens community with a simplification of the community and a decrease in its diversity in comparison to CON and CHI diets. It has been shown that the structure of the methanogens population can be affected by the protozoal community (Belanche, de la Fuente and Newbold 2014; 2015); however, our CCA indicated that this shift could be due to differences in pH and total VFA concentrations in the vessels. This observation suggests that the second hypothesis better explained the antimethanogenic effect of IVY based on the substitution of certain methanogens groups from the *Methanomassilicocaaceae* family (*Groups 3a, 9 and 12)* by others theoretically less active (i.e. *Methanobrevibacter*). Recent work, using DNA and RNA derived 16S RNA analysis (Kang *et al.*^2013^), has shown that *Methanobrevibacter* species despite being numerically predominant only contributed to a third of the RNA-derived *mcrA* sequences. On the contrary, Poulsen *et al.* (2013) reported in a meta-transcriptomic survey that the contribution of *Thermoplasmata*, class to which the *Methanomassilicocaaceae* family belongs, to methane formation might be underestimated by its numerical abundance in cows fed rapeseed oil. Therefore, our results revealed that this specific antimicrobial activity against certain rumen methanogens is likely to be the main driver to decrease rumen methanogenesis in IVY diets. The direct measurement of the concentration of metabolic hydrogen in the vessels could have helped to confirm this later hypothesis.

### Nitrogen metabolism and microbial protein synthesis

Chitin and chitosan have been proposed as novel N-sources for ruminants due to their high N-content (El-Seed *et al.*^2003^). Moreover, chitosan has been demonstrated to be highly digestible (88%–99%) in chickens and rabbits (Hirano *et al.*^1990^), but its digestibility in the rumen is still controversial (El-Seed *et al.*^2003^). Pure CHI used in this experiment had an N-content of 65 g kgDM^−1^ representing an extra N-supply of (+10.8%) to the vessels in form of ammine groups. CHI had similar digestibility to the entire diet since feed disappearance was unaffected by CHI supplementation. Chitosan deamination has been described as an initial step in chitosan degradation by free-living bacteria (Beier and Bertilsson 2013). Therefore, the degradation of amine groups (R-NH_2_) into ammonia (NH_3_) may explain the observed tendency for an increased ammonia concentration (+51%) and ammonia-N overflow in CHI diets. This observation agrees with the increase in rumen ammonia reported in steers fed chitosan (Araujo *et al.*^2015^), but disagrees with those reported for sheep (Goiri, Oregui and Garcia-Rodriguez 2010). The raised concentrations of ammonia observed in our experiment were likely due to an extra supply of ammonia from CHI and low ammonia uptake by the microbes rather than to increased feed proteolysis. Indeed, bacteria fed with CHI had lower ^15^N enrichment than CON as a result of their trend towards a lower ammonia uptake (63% of the total N uptake) or a direct incorporation of amine groups from CHI. Low bacterial ^15^N enrichment could be due to a low bacterial protein breakdown (Belanche *et al.*^2011^) leading to an improved efficiency of protein utilization in vessels fed CHI diets. This improvement was observed in terms of microbial protein flow (+14%) and efficiency of synthesis (g microbial N kgDOM^−1^).

A number of studies have reported contradictory results about the effect of saponins on rumen N metabolism depending on the type of saponins considered and its dose (Patra and Saxena 2009a,b). Dipeptide degradation is considered the limiting factor in rumen proteolysis since dipeptidyl peptidase activity occurs only in very few bacterial species, with *Prevotella ruminicola* by far the most important (Wallace 1996). Therefore, the presence of high numbers of *Prevotella* in vessels fed IVY should promote a rapid degradation of dipeptides into free amino acids and ammonia. Since small peptides are growth factors for many bacterial species (Atasoglu, Newbold and Wallace 2001), its degradation had implications in terms of rumen N metabolism; low availability of free peptides and amino acids forced rumen bacteria to almost exclusively use ammonia as N source in IVY diets (representing 99% of the total N uptake) and ammonia concentrations dropped to values below the concentration required to maximize microbial growth *in vitro* (5 mg dL^−1^) (Griswold *et al.*^2003^). As a result, IVY had a negative impact on microbial protein synthesis (–10%) and on the efficiency of synthesis (–15%). Previous work has also reported that use of saponins in high concentrations have generally adverse effects on microbial protein synthesis (Patra and Saxena 2009a,,b). Moreover, a diet-dependent response to alfalfa saponins on bacterial protein synthesis has been observed (Lu and Jorgensen 1987) and should be further investigated for IVY.

## IMPLICATIONS

This paper revealed that a multiomics approach based on a detailed characterization of the rumen microbiome coupled with an integrated description of the microbial functions and environmental variables is vital to understand the effect and mode of action of new feed additives. Here, we demonstrated that CHI and IVY have a similar ability to decrease methane emissions *in vitro* (by ∼42%) but they differed remarkably in their mode of action: the antimicrobial properties of CHI caused a profound change in the structure of the bacterial community and shifted the fermentation pattern towards propionate production which explained most of the observed decrease in methane emissions. The high amylase activity observed fermenters fed CHI suggested that CHI could also be partially hydrolyzed and used as substrate by rumen microbes. On the contrary, IVY had negligible nutritional properties promoting only minor changes in the fermentation pattern and bacterial community. Instead, IVY had a specific antimicrobial effect of IVY against methanogens which was considered its main antimethanogenic mechanism. Our findings showed that CHI and IVY had promising *in vitro* results as feed additives to modulate rumen function; thus, further *in vivo* research is needed to determine the optimum doses which maintain low methane emissions and also prevent the negative impacts of CHI and IVY on rumen function and ruminants’ metabolism.

## Supplementary Material

Supplementary Data
