# Supplementary material for: Effects and mode of action of chitosan and ivy fruit saponins on the microbiome, fermentation and methanogenesis in the rumen simulation technique
Source: FEMS Microbiol Ecol. 2015 Dec 16;92(1):fiv160. doi: 10.1093/femsec/fiv160 (PMC5831848; doi:10.1093/femsec/fiv160)
Supplement: Supplementary Data [file fiv160_supplementary_data.zip › Supplemental_Material.docx]

**ONLINE SUPPORTING MATERIAL**

**Supplemental Table 1**. Ingredients and chemical composition of the experimental diets.

| **Diets^1^** | **CON** | **CHI** | **IVY** |
| --- | --- | --- | --- |
| **Ingredients (g kg^-1^)** |  |  |  |
| Alfalfa hay | 300 | 285 | 285 |
| Grass hay | 200 | 190 | 190 |
| Barley | 300 | 285 | 285 |
| Corn | 120 | 114 | 114 |
| Soya bean meal | 77 | 73 | 73 |
| Additive | 0 | 50 | 50 |
| Vitamin premix^1^ | 3 | 3 | 3 |
| **Composition (g kg^-1^)** | |  |  |
| Organic matter | 949 | 965 | 950 |
| Nitrogen | 20.5 | 22.8 | 19.7 |
| Carbon | 405 | 403 | 413 |
| Neutral detergent fibre | 370 | 409 | 342 |
| Acid detergent fibre | 211 | 198 | 191 |

^1^ Rumins Cattle GP (Rumeco Ltd, UK.) Declared composition: Ca 240, P 20, Mg 50, Na 80, Se 0.03, Co 0.09, I 0.4, Mn 3, Zn 4 and Cu 1.5 g kg^-1^, retinol 4×10^5^, cholecalciferol 8×10^4^ and alphatocopherol 10^3^ IU kg^-1^

**Supplemental Table 2**. Primers used for quantitative PCR and Ion-Torrent Next Generation Sequencing.

| **Target** | **Author** | **Forward Primer** | **Reverse Primer** | **T^a^** | | **Amplicon (bp)** |
| --- | --- | --- | --- | --- | --- | --- |
| Quantitative PCR |  |  |  | |  |  |
| Total bacteria | ([Maeda *et al.*, 2003](#_ENREF_9)) | GTGSTGCAYGGYTGTCGTCA | ACGTCRTCCMCACCTTCCTC | | 61 | 150 |
| Total protozoa | ([Sylvester *et al.*, 2004](#_ENREF_14)) | GCTTTCGWTGGTAGTGTATT | CTTGCCCTCYAATCGTWCT | | 55 | 223 |
| Anaerobic fungi | ([Denman & McSweeney, 2006](#_ENREF_6)) | GAGGAAGTAAAAGTCGTAACAAGGTTTC | CAAATTCACAAAGGGTAGGATGATT | | 62 | 120 |
| Methanogens | ([Denman *et al.*, 2007](#_ENREF_7)) | TTCGGTGGATCDCARAGRGC | GBARGTCGWAWCCGTAGAATCC | | 56 | 140 |
| Ion Torrent NGS |  |  |  | |  |  |
| Bacterial primers | ([Spear *et al.*, 2008](#_ENREF_13)) | AGAGTTTGATCMTGGCTCAG | CTGCTGCCTYCCGTA | | 58 | 348 |
| Bacterial Adaptors |  | CCATCTCATCCCTGCGTGTCTCCGACTCAG | CCTCTCTATGGGCAGTCGGTGAT | |  |  |
| Methanogens primers | ([Wright & Pimm, 2003](#_ENREF_15)) | GCTCAGTAACACGTGG | GWATTACCGCGGCKGCTG | | 58 | 433 |
| Methanogens adaptors |  | CCATCTCATCCCTGCGTGTCTCCGACTCAG | CCTCTCTATGGGCAGTCGGTGAT | |  |  |

**
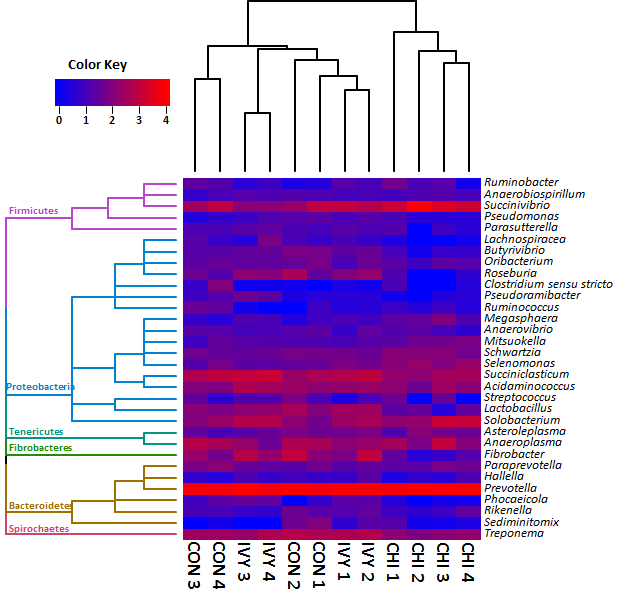
**

**Supplemental Figure 1.** Heat map describing the effect of supplementing a control diet (CON) with chitosan (CHI) or ivy fruit saponins (IVY) on the structure of the rumen bacterial community at the genus level in the Rusitec system. Dendrograms are based on the UPGMA clustering of the Bray-Curtis distances. The total number of reads per sample was log transformed and minor genera were discarded (<1%). Cows used as inoculum are indicated in numbers.

**
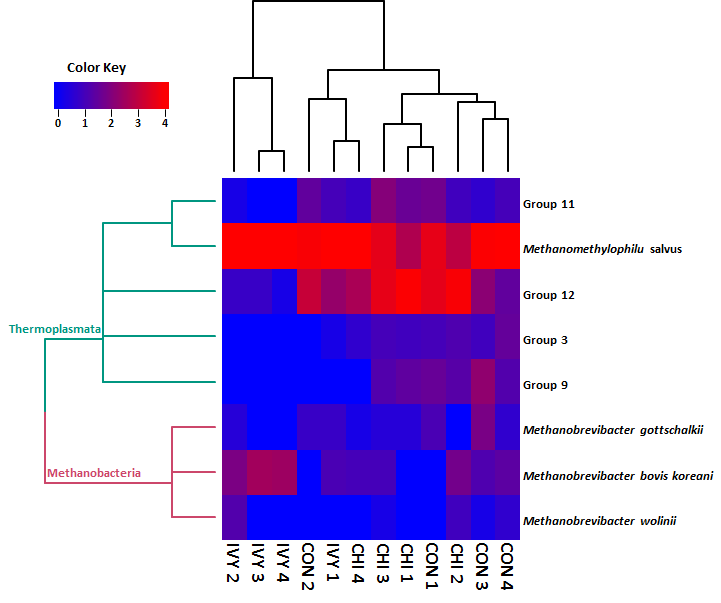
**

**Supplemental Figure 2.** Heat map describing the effect of supplementing a control diet (CON) with chitosan (CHI) or ivy fruit saponins (IVY) on the structure of the rumen methanogens community at the species level in the Rusitec system. Dendrograms are based on the UPGMA clustering of the Bray-Curtis distances. The total number of reads per sample was log transformed. Cows used as inoculum are indicated in numbers.

**Supplemental Table 3.** Effect of supplementing a control diet (CON) with chitosan (CHI) or ivy fruit saponins (IVY) on the relative abundance of the main bacteria at phylum, family and genus level in the Rusitec system.

| **Phylum** | **Family** | | **Genus** | | | | **CON** | **CHI** | **IVY** | **SED** | **Adjusted**  **P-value** |
| --- | --- | --- | --- | --- | --- | --- | --- | --- | --- | --- | --- |
| *Actinobacteria* | | |  | | | | 0.94 | 1.03 | 1.21 | 0.134 | 0.198 |
|  | *Bifidobacteriaceae* | | *Bifidobacterium* | | | | 0.46 | 0.58 | 0.78 | 0.272 | 0.558 |
|  | *Coriobacteriaceae* | | |  | | | 0.79 | 0.89 | 1.05 | 0.091 | 0.123 |
|  |  | | *Eggerthella* | | | | 0.40 | 0.00 | 0.76 | 0.207 | 0.058 |
|  |  | | *Olsenella* | | | | 0.54 | 0.88 | 0.74 | 0.120 | 0.121 |
| *Bacteroidetes* | | |  | | | | 4.10^b^ | 4.24^a^ | 4.15^ab^ | 0.043 | 0.048 |
|  | *Bacteroidales* | | *Phocaeicola* | | | | 0.85^ab^ | 0.30^b^ | 1.55^a^ | 0.238 | 0.023 |
|  | *Flammeovirgaceae* | | | | |  | 2.72 | 1.21 | 1.94 | 0.398 | 0.069 |
|  |  | | *Sediminitomix* | | | | 1.05 | 0.62 | 0.56 | 0.376 | 0.474 |
|  | *Porphyromonadaceae* | | | |  | | 0.58 | 0.45 | 0.35 | 0.182 | 0.527 |
|  | *Prevotellaceae* | | | |  | | 4.02 | 4.07 | 4.09 | 0.043 | 0.437 |
|  |  | | *Hallella* | | | | 0.87 | 0.82 | 1.16 | 0.204 | 0.341 |
|  |  | | *Paraprevotella* | | | | 1.89 | 1.69 | 1.59 | 0.146 | 0.272 |
|  |  | | *Prevotella* | | | | 4.00 | 4.06 | 4.07 | 0.044 | 0.399 |
|  |  | | *Others Prevotellaceae* | | | | 2.62^ab^ | 2.43^b^ | 2.69^a^ | 0.062 | 0.036 |
|  | *Rikenellaceae* | | *Rikenella* | | | | 1.41 | 1.09 | 1.18 | 0.254 | 0.527 |
|  | *Others* | | | |  | | 3.14^b^ | 3.71^a^ | 3.18^b^ | 0.121 | 0.023 |
| *Fibrobacteres* | *Fibrobacteraceae* | | *Fibrobacter* | | | | 2.44^a^ | 1.16^b^ | 2.65^a^ | 0.397 | 0.048 |
| *Firmicutes* | |  |  | | | | 3.86^a^ | 3.53^b^ | 3.84^a^ | 0.098 | 0.048 |
|  | *Acidaminococcaceae* | | | |  | | 2.96^b^ | 2.71^c^ | 3.24^a^ | 0.085 | 0.023 |
|  |  | | *Acidaminococcus* | | | | 2.26 | 2.24 | 2.60 | 0.182 | 0.238 |
|  |  | | *Succiniclasticum* | | | | 2.82^b^ | 2.52^c^ | 3.12^a^ | 0.092 | 0.023 |
|  | *Clostridiaceae* | | | |  | | 1.35 | 0.86 | 0.76 | 0.363 | 0.288 |
|  |  | | | | *Sensu stricto* | | 0.86 | 0.46 | 0.35 | 0.482 | 0.570 |
|  | *Erysipelotrichaceae* | | | |  | | 2.26 | 2.84 | 2.79 | 0.172 | 0.069 |
|  |  | | *Erysipelotrichaceae incertae* | | | | 1.08 | 0.34 | 0.19 | 0.254 | 0.055 |
|  |  | | *Solobacterium* | | | | 2.20 | 2.80 | 2.78 | 0.175 | 0.052 |
|  | *Eubacteriaceae* | | | |  | | 0.94^a^ | 0.40^b^ | 1.23^a^ | 0.148 | 0.023 |
|  |  | | *Eubacterium* | | | | 0.27 | 0.00 | 0.48 | 0.164 | 0.117 |
|  |  | | *Pseudoramibacter* | | | | 0.90^a^ | 0.40^b^ | 1.19^a^ | 0.143 | 0.029 |
|  | *Lachnospiraceae* | | | |  | | 2.72 | 2.50 | 2.78 | 0.134 | 0.226 |
|  |  | | *Butyrivibrio* | | | | 1.71^a^ | 0.81^b^ | 1.63^a^ | 0.218 | 0.034 |
|  |  | | *Catonella* | | | | 0.66 | 0.78 | 0.98 | 0.314 | 0.664 |
|  |  | | *Coprococcus* | | | | 0.60^a^ | 0.00^b^ | 0.68^a^ | 0.142 | 0.029 |
|  |  | | *Lachnospiracea incertae* | | | | 1.12 | 0.19 | 1.19 | 0.316 | 0.064 |
|  |  | | *Oribacterium* | | | | 1.72 | 1.36 | 1.55 | 0.173 | 0.272 |
|  |  | | *Pseudobutyrivibrio* | | | | 0.59^a^ | 0.12^b^ | 0.83^a^ | 0.082 | 0.003 |
|  |  | | *Roseburia* | | | | 1.90^a^ | 0.52^b^ | 2.29^a^ | 0.425 | 0.036 |
|  |  | | *Syntrophococcus* | | | | 0.68^b^ | 0.47^b^ | 1.28^a^ | 0.165 | 0.030 |
|  | *Lactobacillaceae* | | *Lactobacillus* | | | | 2.28^a^ | 1.35^b^ | 2.49^a^ | 0.202 | 0.023 |
|  | *Ruminococcaceae* | | | |  | | 1.85 | 1.67 | 1.43 | 0.156 | 0.133 |
|  |  | | *Ruminococcus* | | | | 1.09 | 0.81 | 0.40 | 0.382 | 0.341 |
|  | *Streptococcaceae* | | *Streptococcus* | | | | 1.43 | 0.87 | 1.12 | 0.521 | 0.588 |
|  | *Veillonellaceae* | | | |  | | 2.26^b^ | 2.72^a^ | 2.25^b^ | 0.063 | 0.002 |
|  |  | | *Anaerovibrio* | | | | 1.45 | 1.19 | 1.22 | 0.158 | 0.341 |
|  |  | | *Megasphaera* | | | | 0.82^b^ | 1.62^a^ | 1.22^ab^ | 0.146 | 0.029 |
|  |  | | *Mitsuokella* | | | | 1.33^b^ | 1.79^a^ | 1.36^b^ | 0.121 | 0.036 |
|  |  | | *Schwartzia* | | | | 1.84^b^ | 2.13^a^ | 1.74^b^ | 0.078 | 0.029 |
|  |  | | *Selenomonas* | | | | 1.65^b^ | 2.31^a^ | 1.74^b^ | 0.149 | 0.034 |
|  | *Others* | | | |  | | 3.69 | 2.85 | 3.49 | 0.282 | 0.109 |
| *Proteobacteria* | | |  | | | | 2.91^b^ | 3.62^a^ | 2.89^b^ | 0.255 | 0.048 |
|  | *Desulfovibrionaceae* | | | |  | | 1.17 | 0.88 | 1.32 | 0.144 | 0.111 |
|  | *Pseudomonadaceae* | | *Pseudomonas* | | | | 1.09 | 0.84 | 1.25 | 0.169 | 0.174 |
|  | *Succinivibrionaceae* | | | |  | | 2.84 | 3.61 | 2.68 | 0.283 | 0.081 |
|  |  | | *Anaerobiospirillum* | | | | 1.17 | 1.25 | 1.25 | 0.139 | 0.830 |
|  |  | | *Ruminobacter* | | | | 0.99 | 1.20 | 1.09 | 0.417 | 0.882 |
|  |  | | *Succinivibrio* | | | | 2.81 | 3.60 | 2.64 | 0.295 | 0.064 |
|  | *Sutterellaceae* | | *Parasutterella* | | | | 1.24 | 0.78 | 1.44 | 0.239 | 0.123 |
|  | *Others* | | | |  | | 1.77 | 0.94 | 1.78 | 0.465 | 0.253 |
| *Spirochaetes* | | |  | | | | 2.76^a^ | 2.26^b^ | 2.81^a^ | 0.125 | 0.048 |
|  | *Spirochaetaceae* | | *Treponema* | | | | 2.76^a^ | 2.26^b^ | 2.81^a^ | 0.125 | 0.030 |
| *Synergistetes* | | |  | | | | 0.73 | 1.16 | 0.58 | 0.211 | 0.087 |
|  |  | | *Jonquetella* | | | | 0.73^a^ | 0.08^b^ | 0.42^ab^ | 0.139 | 0.034 |
|  |  | | *Pyramidobacter* | | | | 0.00^b^ | 1.14^a^ | 0.27^b^ | 0.182 | 0.023 |
| *Tenericutes* | *Anaeroplasmataceae* | | | |  | | 2.86^a^ | 2.68^ab^ | 2.38^b^ | 0.137 | 0.048 |
|  |  | | *Anaeroplasma* | | | | 2.82 | 2.52 | 2.23 | 0.206 | 0.121 |
|  |  | | *Asteroleplasma* | | | | 1.67 | 1.84 | 1.77 | 0.197 | 0.766 |
| Unlassified | | |  | | | | 3.83^a^ | 3.14^b^ | 3.75^a^ | 0.215 | 0.048 |

^1^The total number of reads per sample was log-transformed and minor genera were discarded. Within a raw means without a common superscript differ (*P* < 0.05).

| **Family** | **Genus** | **Species** | **CON** | **CHI** | **IVY** | **SED** | **Adjusted**  **P-value** |
| --- | --- | --- | --- | --- | --- | --- | --- |
| *Methanomassiliicoccaceae* | | | 3.06 | 3.06 | 3.05 | 0.005 | 0.060 |
|  | *Group 11* |  | 2.95 | 2.55 | 3.04 | 0.157 | 0.066 |
|  |  | *Methanomethylophilus alvus* | 2.94 | 2.52 | 3.04 | 0.162 | 0.041 |
|  |  | *Others* | 1.00 | 1.09 | 0.29 | 0.242 | 0.062 |
|  | *Group 12* |  | 1.98^b^ | 2.69^a^ | 0.87^c^ | 0.198 | 0.001 |
|  | *Group 3a* |  | 1.03 | 0.86 | 0.15 | 0.295 | 0.052 |
|  | *Group 9* |  | 0.99 | 0.80 | 0.00 | 0.368 | 0.077 |
| *Methanobacteriaceae* | | | 1.12 | 0.96 | 1.63 | 0.230 | 0.060 |
|  | *Methanobrevibacter* | | 1.12 | 0.96 | 1.63 | 0.230 | 0.060 |
|  |  | *M bovis koreani* | 0.52 | 0.77 | 1.55 | 0.272 | 0.062 |
|  |  | *M gottschalkii* | 0.92 | 0.31 | 0.29 | 0.230 | 0.071 |
|  |  | *M wolinii* | 0.23 | 0.27 | 0.25 | 0.266 | 0.986 |

**Suplemental Table 4.** Effect of supplementing a control diet (CON) with chitosan (CHI) or ivy fruit saponins (IVY) on the relative abundance of the main archaea at family, genus and species level in the Rusitec system.

^1^The total number of reads per sample was log-transformed. Within a raw means without a common superscript differ (*P* < 0.05).
